# The relationship between school innovation climate and teacher innovation behavior in higher vocational colleges: the chain mediating role of teacher job crafting and creative self-efficacy

**DOI:** 10.3389/fpsyg.2026.1924843

**Published:** 2026-09-14

**Authors:** Xianhui Jin, Pan Tian, Xinyi Yang

**Affiliations:** 1 School of Humanities, Xuchang Vocational Technical College, Xuchang, Henan, China; 2 School of Medical and Health Sciences, Xuchang Vocational Technical College, Xuchang, Henan, China

**Keywords:** creative self-efficacy, higher vocational college, job crafting, school innovation climate, teacher innovation behavior

## Abstract

**Background:**

Teacher innovation behavior is essential to improving instructional quality and talent cultivation in higher vocational education, yet the psychological mechanisms linking school innovation climate to such behavior remain insufficiently understood. Drawing on the Job Demands-Resources framework, this study examined whether job crafting and creative self-efficacy sequentially mediate the relationship between school innovation climate and teacher innovation behavior.

**Methods:**

A questionnaire survey was conducted among 603 teachers from higher vocational colleges between May and June 2026 through the Wenjuanxing platform. School innovation climate, job crafting, creative self-efficacy, and teacher innovation behavior were measured using established scales. Data were analyzed with SPSS 27.0 and AMOS 27.0 through descriptive statistics, reliability and validity testing, common method bias assessment, correlation analysis, and mediation analysis.

**Results:**

The results showed that school innovation climate was positively associated with teacher innovation behavior. Job crafting and creative self-efficacy each served as significant mediators, and they also formed a significant sequential mediation pathway. The indirect effects of job crafting and creative self-efficacy on the relationship between school innovation climate and teacher innovation behavior were 0.260 and 0.097, respectively, and the sequential indirect effect of job crafting and creative self-efficacy was estimated at 0.074. These three indirect effects accounted for 43.16, 16.18, and 12.31% of the total effect, respectively.

**Conclusion:**

These findings suggest that a supportive school innovation climate is positively associated with teacher innovation behavior, with job crafting and creative self-efficacy representing significant indirect pathways in this relationship.

## 1 Introduction

In the context of the high-quality development of vocational education and the continuous advancement of teaching reforms, talent cultivation in higher vocational colleges has placed increasingly stringent demands on classroom teaching quality and teachers’ instructional approaches. As the direct implementers of teaching activities, teachers’ capacity to proactively update teaching content, improve teaching methods, and flexibly apply teaching resources is closely associated with the enhancement of teaching quality (Andersson and Köpsén, 2017; Zhou et al., 2022). Teacher innovation behavior refers to teachers’ behavioral tendency to actively explore new teaching philosophies during teaching practice and adopt diversified instructional methods or technologies to enhance student engagement and optimize the teaching process (Anderson et al., 2014). For higher vocational teachers, teacher innovation behavior is not only associated with the improvement of classroom teaching quality and students’ practical abilities, but also represents an important manifestation of teachers’ adaptation to vocational education reform and enhancement of teaching vitality (He and Jen, 2025; Lin et al., 2022; Zhou et al., 2022). Compared with more general educational settings, higher vocational colleges place stronger emphasis on applied skill development, industry-education integration, practical training, and the incorporation of digital and occupational technologies into teaching (Ma et al., 2025; Zhu et al., 2025). These institutional demands require teachers to coordinate classroom instruction with practice-based learning, update teaching content in response to changes in industry and technology, and continuously adjust instructional methods to meet the needs of skilled talent cultivation. Accordingly, teacher innovation behavior is especially salient in higher vocational colleges because teachers must repeatedly adapt curricula, teaching strategies, and practical training arrangements to the changing requirements of vocational education reform. Exploring the formation mechanism of teacher innovation behavior in higher vocational colleges holds important theoretical significance and practical value.

Existing research shows that teacher innovation behavior is closely related to personal factors and school organizational environmental factors. In terms of individual factors, variables including creative self-efficacy, job crafting, work engagement, AI literacy, and knowledge sharing have been shown to significantly correlate with teacher innovation behavior (Huang et al., 2022; Shi et al., 2023; Siregar et al., 2025; Zhao and Huang, 2025; Zheng et al., 2024). Regarding the organizational milieu, elements encompassing principal leadership, school innovation climate, and perceived support have likewise been identified as antecedents of teacher innovation behavior (Al-Mahdy and Elwakil, 2026; Alshuhumi et al., 2025; Bao, 2024; Liu et al., 2016; Zhao and Huang, 2025). Teacher innovation behavior is associated not only with teachers’ ability beliefs, proactive work adjustments, and professional engagement, but also with the supportive environment and innovation climate provided by schools. Among these factors, the school innovation climate, as an important contextual resource in the school organizational environment, reflects support for teachers in carrying out teaching reforms, experimenting with new teaching methods, and sharing innovative resources, and is positively associated with teacher innovation behavior (Anderson and West, 1998; Bao, 2024). However, explaining teacher innovation behavior solely from the perspective of the organizational environment remains insufficient. Teacher innovation behavior does not simply constitute a passive reaction to external support; instead, it more plausibly emerges from educators proactively modifying their work approaches and reinforcing their innovation-related convictions when contextual resources activate such involvement. Job crafting emphasizes individuals’ proactive adjustments to their work tasks, work cognition, and work relationships to improve person–job fit and access to work-related resources (Slemp and Vella-Brodrick, 2013; Zheng et al., 2024). Creative self-efficacy, in contrast, reflects teachers’ beliefs in their capability to carry out creative teaching activities, design innovative courses, and overcome challenges in instructional innovation (Lin and Chiou, 2008; Shi et al., 2023). These two constructs capture different but potentially connected aspects of teachers’ responses to an innovation-supportive work environment. When teachers actively modify teaching tasks, seek or reorganize work resources, and expand professional interactions, they may encounter more opportunities for instructional experimentation, feedback, and successful teaching experiences. Such work experiences are closely relevant to teachers’ evaluations of their capability to engage in creative teaching. Accordingly, job crafting and creative self-efficacy may represent interconnected behavioral and psychological processes through which school innovation climate is associated with teacher innovation behavior. Integrating school innovation climate, job crafting, and creative self-efficacy into a unified analytical framework therefore provides a more process-oriented understanding of teacher innovation behavior in higher vocational colleges.

The innovative practice of higher vocational teachers is not merely contingent upon whether the institution furnishes a supportive milieu; it is also closely associated with whether teachers proactively adjust their work approaches and whether they possess creative teaching beliefs. Yet the majority of prior investigations have explored the associations among school innovation climate, job crafting, creative self-efficacy, and teacher innovation behavior via isolated pathways or bivariate correlations, thereby falling short of comprehensively revealing the synergistic mechanism governing these variables within a unified analytical framework. Two issues therefore warrant further examination. First, although school innovation climate has been associated with job crafting and creative self-efficacy separately, less is known about whether teachers’ proactive work adjustments and creative capability beliefs may operate as connected processes linking an innovation-supportive environment with teacher innovation behavior. Second, limited attention has been paid to these relationships among higher vocational teachers, whose work is characterized by strong practice orientation, industry–education integration, and continuing adaptation to technological and occupational change. Based on the Job Demands-Resources model (Bakker and Demerouti, 2007), the present study addresses these gaps by integrating school innovation climate as an organizational job resource, job crafting as a proactive work-adjustment process, and creative self-efficacy as a personal psychological resource within a serial mediation framework. Rather than examining these variables separately, the study tests whether school innovation climate is indirectly associated with teacher innovation behavior through job crafting and subsequently creative self-efficacy among higher vocational teachers. These outcomes furnish conceptual foundations for elucidating the generative processes underlying teacher innovation behavior within vocational institutions, while simultaneously yielding actionable guidance for optimizing innovation-conducive settings and cultivating educators’ proactive dispositions alongside creative pedagogical convictions.

## 2 Review and hypothesis

### 2.1 Theoretical basis

The Job Demands-Resources model (JD-R model) was originally proposed by Demerouti et al. (2001) and subsequently developed by Bakker and Demerouti (2007). According to the framework, there are two main categories of job characteristics: resources and demands. Demands are aspects of a profession that need ongoing mental and physical effort from employees. Conversely, resources are task-related, social, and organizational assets. These assets serve multiple functions, including facilitating the achievement of work objectives, reducing various costs, and fostering individual development and career progress. According to additional research, personal resources, such as self-efficacy, optimism and psychological resilience, are important parts of the JD-R model and are closely related to people’s good working conditions and work performance (Bakker and Demerouti, 2007; Xanthopoulou et al., 2007). This theoretical framework not only focuses on the external working environment, but also emphasizes how individuals can develop positive work adaptation and behavior with the support of sufficient resources.

Studies on burnout, work engagement, job crafting, and inventive behavior have made substantial use of the JD-R model (Bakker and Demerouti, 2007; Slemp and Vella-Brodrick, 2013; Tims et al., 2012). In this study, school innovation climate can be regarded as an organizational-level job resource, which provides an environmental foundation for teachers to experiment with new ideas, obtain innovative support, and share teaching resources (Anderson and West, 1998; Bao, 2024). When teachers perceive that the school environment supports innovation, they are more likely to proactively adjust teaching tasks, reinterpret the value of their teaching work, and expand collaborative relationships with colleagues, thereby exhibiting a higher level of job crafting. Job crafting is associated with adjustments to teaching methods, successful experiences, teachers’ sense of control, and higher levels of creative self-efficacy, which is an important personal resource (Lin and Chiou, 2008; Shi et al., 2023). Based on the JD-R model, this study explores the relationship between school innovation climate, job crafting, creative self-efficacy and teacher innovation behavior, taking into account work resources, proactive behavior and personal resources.

### 2.2 School innovation climate and teacher innovation behavior

The school innovation climate denotes the common understanding of school members about the extent to which organizations encourage new ideas, support change-oriented practices, promote resource sharing and promote innovation implementation (Anderson and West, 1998). In the context of higher vocational colleges, a positive school innovation climate is reflected in institutional support for teaching reform, technology integration, and instructional method innovation, as well as in the institutional guarantees, peer collaboration, and resource support available to teachers when they explore new teaching concepts (Bao, 2024; Zhao and Huang, 2025). According to the JD-R model, school innovation climate may be thought of as an organizational-level job resource that provides instructors with outside assistance to engage in creative practices and carry out instructional reform responsibilities (Bakker and Demerouti, 2007; Bao, 2024). Existing studies suggest that an innovation-oriented school climate is positively related to teacher innovation behavior (Al-Mahdy and Elwakil, 2026; Bao, 2024). When higher vocational teachers perceive that the school encourages innovation, respects teaching exploration, and provides resource support, they are more inclined to form positive attitudes toward teaching reform, experiment with new teaching ideas, and integrate digital technologies and other instructional tools to optimize classroom teaching processes (Al-Mahdy and Elwakil, 2026; Bao, 2024; Cai and Tang, 2021). For example, Bao (2024) incorporated school innovation climate into a model of teacher innovation behavior and found that school innovation climate functions as an important environmental mechanism linking school leadership contexts to teacher innovation behavior. Al-Mahdy and Elwakil (2026) also reported in their study on teacher innovation behavior that a strong association exists between innovative school climate and teacher innovation behavior. Thus, a stronger school innovation climate may be positively associated with a greater tendency among higher vocational teachers to exhibit teacher innovation behavior.

### 2.3 The mediating role of job crafting

In the education industry, job crafting shows that teachers take the initiative to improve the arrangement of teaching tasks, reinterpret the value of teaching work, and optimize teaching practice through colleague cooperation, experience exchange and resource sharing (Aulén et al., 2024; Leana et al., 2009). School innovation climate, as organizational innovation support perceived by teachers, includes encouragement of new ideas, institutional support for teaching reform, and resource sharing and collaboration opportunities. Theoretically, such a climate creates favorable conditions for teachers to take initiative in their work and is linked to job crafting (Miao et al., 2023; Xu et al., 2025). Empirical research also supports this relationship: Xu et al. (2025) found that school climate is a significant positive predictor of teacher job crafting, indicating that a supportive school environment is conducive to teachers’ adjustment of work content and methods. Teachers’ attempts at teaching and job crafting may have a contextual basis in the school innovation climate, which is a significant factor that represents innovation support. In addition, Miao et al. (2023) found that an innovative organizational climate can enhance the relationship between organizational resources and employee job crafting, which shows that the working environment that encourages innovation provides important environmental conditions for people to take the initiative to reshape their work. Job crafting is not only a proactive behavior of teachers, but also closely associated with teacher innovation behavior (Huang et al., 2022; Ninković et al., 2025). For instance, Huang et al. (2022) found in a study of Chinese teachers that teacher job crafting was significantly associated with creative teaching performance, suggesting that teachers’ proactive modification of work tasks and allocation of resources are closely associated with creative teaching practices. Within the JD-R model, school innovation climate functions as a job resource linked to teachers’ job crafting behaviors, while job crafting, as a proactive behavior, is related to personal resources and innovative teaching practices (Bakker and Demerouti, 2007, 2017). In summary, a positive school innovation climate may be indirectly associated with higher vocational teachers’ teacher innovation behavior through enhanced levels of teacher job crafting.

### 2.4 The mediating role of creative self-efficacy

In teaching contexts, creative self-efficacy is mainly reflected in teachers’ confidence in designing innovative curricula, applying creative instructional strategies, encouraging students’ creative behaviors, and addressing challenges in teaching innovation (Lin and Chiou, 2008). School innovation climate, as an organizational-level job resource, provides teachers with innovation support, resource sharing, and openness to change, and corresponds to higher levels of creative self-efficacy (Liu and Zaman, 2025; Liu et al., 2022). Previous research has also supported this association. For example, Liu and Zaman (2025) looked at Chinese physical education instructors and discovered that teacher innovation behavior was related to both the school innovation climate and the teachers’ creative self-efficacy. According to this research, teachers’ self-confidence in their creative skills is strongly correlated with an innovative and creative educational environment. Liu et al. (2022) also reported that school innovation climate is linked to a stronger association between professional learning community and teachers’ creative self-efficacy. School environments that emphasize innovation offer important contextual conditions for strengthening teachers’ creative beliefs. For educators, creative self-efficacy constitutes a vital psychological asset and strongly correlates with their tendency to engage in innovative practices (Chen, 2024; Liu and Zaman, 2025; Shi et al., 2023). For example, Shi et al. (2023) found that teachers’ creative teaching self-efficacy is significantly associated with creative teaching behavior, and teachers’ beliefs about their own creative teaching ability are an important psychological resource for understanding teacher innovation behavior. Teacher innovation behavior is linked to creative self-efficacy, which is also positively associated with school innovation climate when the latter is regarded as a work-related resource under the JD-R model. Creative self-efficacy further operates as an internal psychological resource that is closely associated with innovative actions (Bakker and Demerouti, 2017; Xanthopoulou et al., 2007). Taken together, within higher vocational colleges, a supportive climate for school innovation may be indirectly connected to teacher innovation behavior by strengthening their creative self-efficacy.

### 2.5 The chain mediating effect of job crafting and creative self-efficacy

Job crafting and creative self-efficacy may be closely related (Huang et al., 2022; Huang et al., 2023). Job crafting involves teachers’ proactive adjustment of teaching tasks, reconceptualization of teaching work, and optimization of teaching practice through colleague collaboration and resource sharing (Slemp and Vella-Brodrick, 2013; Tims et al., 2012). By adjusting teaching tasks, professional relationships, and available resources, teachers may gain opportunities to experiment with teaching approaches, accumulate successful experiences, and receive professional support and feedback, all of which are relevant to their judgments of creative teaching capability. Huang et al. (2022) further demonstrated that teachers’ job crafting is closely connected with creative teaching performance, indicating an association between proactive adjustments to teaching practice and engagement in creative instructional activities. Such adjustments may therefore provide an experiential basis for more positive judgments of creative teaching capability and link job crafting with creative self-efficacy.

Furthermore, school innovation climate may provide the organizational resource foundation for this chain-like pathway. In the JD-R model, organizational-level job resources not only provide support for individuals to complete work tasks, but are also associated with the tendency to proactively adjust work content, optimize work relationships, and reconstruct work meaning (Bakker and Demerouti, 2007; Xanthopoulou et al., 2007). School innovation climate, as an organizational-level work resource, is manifested in institutional support for new ideas, teaching reform, resource sharing, and innovative practices (Anderson and West, 1998; Bao, 2024). When teachers perceive that the school encourages innovation and provides practical support, they may be more inclined to proactively reshape teaching tasks, work meaning, and professional relationships. These proactive adjustments may expose teachers to additional opportunities for instructional experimentation, resource utilization, and work-related feedback, which are in turn relevant to their judgments of creative teaching capability. Stronger creative self-efficacy is further associated with greater engagement in innovative teaching behavior. Accordingly, the proposed serial pathway proceeds from school innovation climate as an organizational resource, through job crafting as a proactive behavioral response, to creative self-efficacy as an innovation-related personal resource, and ultimately to teacher innovation behavior. This study examines whether job crafting and creative self-efficacy function as possible sequential mediating mechanisms linking school innovation climate to teacher innovation behavior in higher vocational colleges.

### 2.6 Hypothesis

Based on the JD-R model and existing research evidence, school innovation climate may be associated with higher vocational teacher innovation behavior through multiple resource pathways. Specifically, as an organizational-level work resource, school innovation climate may be not only directly related to teacher innovation behavior, but also indirectly associated with such behavior through enhanced levels of teacher job crafting and creative self-efficacy. To examine this mechanism systematically, the present study develops the theoretical model presented in Figure 1 and advances the following hypotheses:

**Figure 1:**
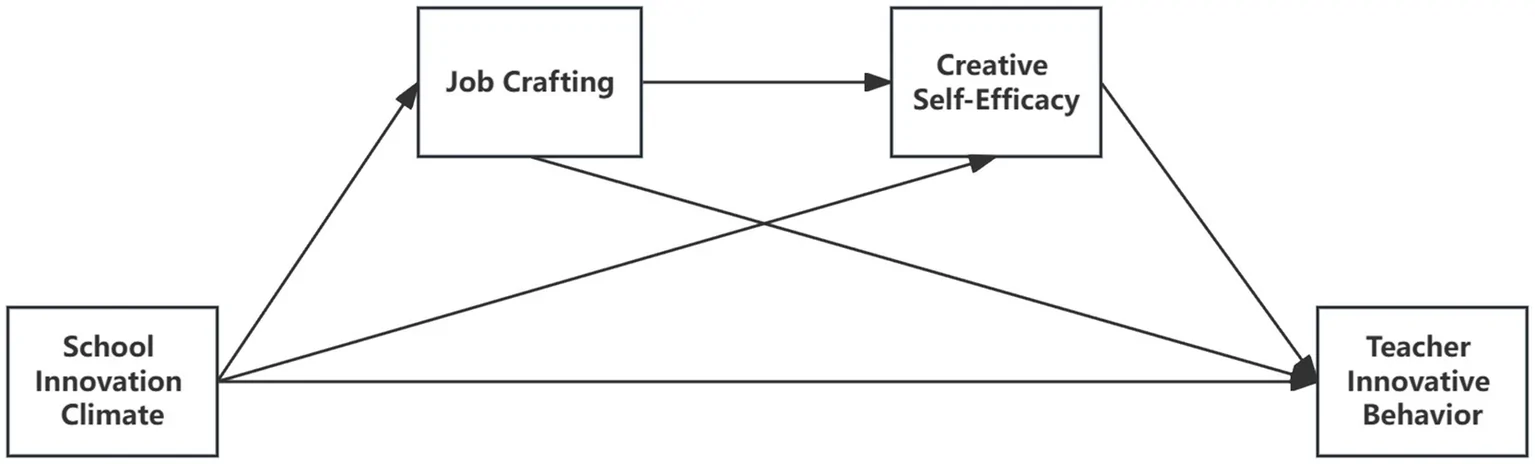
Theoretical model of research.

*H*1: School innovation climate is positively correlated with teacher innovation behavior in higher vocational colleges.

*H*2: Job crafting mediates the relationship between school innovation climate and teacher innovation behavior in higher vocational colleges.

*H*3: Creative self-efficacy mediates the relationship between school innovation climate and teacher innovation behavior in higher vocational colleges.

*H*4: Job crafting and creative self-efficacy play a chain mediating role between school innovation climate and teacher innovation behavior in higher vocational colleges.

## 3 Research method

### 3.1 Sample and data collection

This study conducted an online questionnaire survey among teachers from three higher vocational colleges in Henan Province through the Wenjuanxing platform between May and June 2026. After administrative approval was obtained from the three participating institutions, teacher rosters were acquired, and participants within each institution were randomly selected using random number generation. With the assistance of each institution, teachers were informed of the research purposes, voluntary participation, and principles of data anonymity and confidentiality in the survey settings. Questionnaire links were sent exclusively to randomly selected teachers, with a unique identification code assigned to each questionnaire. Respondents’ identities were strictly controlled, and valid questionnaires were ultimately collected for subsequent statistical analysis.

Sample-size adequacy was assessed using two complementary approaches. First, based on the commonly used respondent-to-item ratio of 10:1 (Boateng et al., 2018; Nunnally, 1978), only the 43 substantive measurement items were considered for sample-size determination; the six demographic questions were excluded, resulting in a minimum required sample size of 430 participants (43 items × 10 respondents). Second, an *a priori* power analysis was conducted using G*Power 3.1. With a medium effect size (f^2^ = 0.15), an alpha level of 0.05, a statistical power of 0.95, and six predictors, the analysis indicated a minimum required sample size of 146 participants (Faul et al., 2009). Therefore, the more conservative criterion of 430 participants was adopted as the minimum sample-size requirement. To account for potentially unusable questionnaires, a total of 642 questionnaires were distributed. After data cleaning, 39 questionnaires were excluded because of missing responses or extreme response patterns (e.g., selecting “strongly agree” or “strongly disagree” for all items). Thus, 603 valid questionnaires were retained for analysis, yielding an effective response rate of 93.93%.

Table 1 presents the background data on all participants: among them, 262 men (43.4%) and 341 women (56.6%) were included. Regarding age, teachers aged 51 and above constituted the largest proportion (33.2%). In terms of professional category, science, engineering, and medicine teachers were the most numerous (41.8%). For teaching experience, teachers with 11–15 years accounted for the highest proportion (27.0%). Educational background was predominantly master’s degree (36.8%) and bachelor’s degree (35.5%). Regarding professional title, teachers with intermediate titles constituted the largest proportion (39.8%). Independent-samples t tests and one-way analyses of variance were conducted to examine whether teacher innovation behavior differed across demographic groups. Significant differences in teacher innovation behavior were observed for gender, professional category, and educational background, whereas differences associated with age, teaching experience, and professional title were not statistically significant. Based on these preliminary demographic comparisons, and to maintain a parsimonious model, gender, professional category, and educational background were included as covariates in the subsequent regression analyses.

**Table 1:** Demographic characteristics of the sample.

Demographic characteristics	Category	Amount	Percentage (%)	*t/F*	*p*
Gender	Men	262	43.4	−2.311	0.021
	Women	341	56.6		
Age	≤30	125	20.7	1.610	0.186
	31–40	127	21.1		
	41–50	151	25.0		
	≥51	200	33.2		
Professional category	Humanities/Economics and Management	165	27.4	4.222	0.015
	Science/Engineering/Medicine	252	41.8		
	Arts/Physical Education	186	30.8		
Teaching experience	1–3	84	13.9	1.702	0.148
	4–6	141	23.4		
	7–10	68	11.3		
	11–15	163	27.0		
	≥16	147	24.4		
Education background	Doctoral	134	22.2	6.902	< 0.001
	Master	222	36.8		
	Bachelor	214	35.5		
	Associate	33	5.5		
Title	Junior	164	27.2	0.228	0.877
	Intermediate	240	39.8		
	Associate senior	112	18.6		
	Full senior	87	14.4		

### 3.2 Measurement instruments

#### 3.2.1 School innovation climate

This study used the School Innovation Climate Scale adapted by Yu (2018) from Amabile (1996) to measure higher vocational college teachers’ perceptions of their school’s support for innovation. The scale includes two dimensions: creative rewards and creative support, with a total of 8 items. Example items include “The school encourages colleagues to come up with creative ideas” and “Teachers who carry out creative teaching activities are recognized.” All items are rated on a 5-point Likert scale (1 = strongly disagree, 5 = strongly agree), with higher scores indicating a more positive perception of the school’s innovation climate. The scale has been validated among Chinese teachers and shows good internal consistency (Chang et al., 2021). In this study, the Cronbach’s α was 0.921, indicating good internal consistency.

#### 3.2.2 Job crafting

This study used the Job Crafting Scale developed by Slemp and Vella-Brodrick (2013) to measure the level of job crafting among higher vocational teachers, with reference to its application among Chinese teachers by Zheng et al. (2024). The scale consists of 15 items across three dimensions: task crafting, cognitive crafting, and relational crafting. Example items include “You introduce new approaches to improve your work,” “You think about how your work gives your life meaning,” and “You make an effort to get to know people better at work.” All items were rated on a 6-point Likert scale (1 = almost never, 6 = always), with higher total scores indicating higher levels of proactive crafting tendencies in teachers’ work. In this investigation, the Cronbach’s α coefficient for this scale was 0.969, demonstrating excellent internal consistency.

#### 3.2.3 Creative self-efficacy

The Creative Self-Efficacy Scale, created by Lin and Chiou (2008), was used in this study to gage higher vocational teachers’ perceptions of their participation in innovative teaching practices. The scale consists of 15 items encompassing three dimensions: positive affirmation, negative self-awareness, and stress-resistance belief. It primarily assesses teachers’ confidence in designing creative teaching activities, stimulating students’ creative behaviors, employing creative problem-solving strategies, and persisting in creative teaching under unfavorable instructional conditions. Items in the negative self-awareness dimension are reverse-worded and were reverse-scored before reliability analysis and CFA. All 15 items, including the reverse-scored items, were modeled as indicators of a single creative self-efficacy construct. All items are rated on a 5-point Likert scale (1 = strongly disagree, 5 = strongly agree), with higher total scores indicating stronger creative self-efficacy. The scale has been validated in Chinese populations and demonstrates good internal consistency (Shi et al., 2023). In the present study, the Cronbach’s α coefficient for this scale was 0.959, indicating satisfactory internal consistency.

#### 3.2.4 Teacher innovation behavior

This study used the Teacher Innovation Behavior Scale developed by Liu et al. (2016) to measure the teaching innovation of higher vocational teachers. The scale consists of 5 items and examines teachers’ tendency to explore new teaching ideas and utilize varied teaching methods or technology to increase student involvement. All items were rated on a 5-point Likert scale (1 = strongly disagree, 5 = strongly agree), with higher total scores indicating higher levels of teaching innovation. This scale has been validated among Chinese populations, with good internal consistency (Zhao and Huang, 2025). The Cronbach’s α for this scale was 0.886, indicating good internal reliability.

### 3.3 Statistical analysis

This study used SPSS 27.0 and AMOS 27.0 to perform the statistical analyses. First, descriptive statistics were applied to examine the distributional characteristics of the study variables. Next, the reliability and validity of the measurement instruments were evaluated. On this basis, confirmatory factor analysis (CFA) was carried out to assess the fit of the measurement model and to detect potential common method bias (CMB). After the measurement properties of the constructs were evaluated, composite scores were calculated for the study variables and used in the subsequent analyses. Hierarchical regression analysis was then used to examine the associations among variables. Finally, the serial indirect associations were examined using Model 6 of the PROCESS macro (Version 4.2). Bootstrap confidence intervals for the indirect associations were estimated using 5,000 resamples. CFA and PROCESS served complementary purposes: CFA was used to evaluate the measurement model, whereas PROCESS was used to estimate the direct and specific indirect associations among the composite scores and to obtain bootstrap confidence intervals for the indirect associations.

## 4 Results

### 4.1 Reliability and validity testing

As reported in Table 2, the reliability of the measurement scales was examined through composite reliability (CR) and Cronbach’s alpha. Following Fornell and Larcker (1981), CR values exceeding 0.70 are regarded as acceptable, and Cronbach’s alpha values above 0.70 also indicate adequate reliability (Leguina, 2015). The results showed that both reliability indices for each scale reached the recommended standards, demonstrating satisfactory internal consistency. Additionally, the average variance extracted (AVE) was calculated to evaluate convergent validity. According to Leguina (2015), an AVE value of 0.50 or higher is generally considered acceptable. Since the AVE values of all constructs were above this threshold, the measurement items showed sufficient convergent validity. The standardized factor loadings for all 43 items are reported in Supplementary Table S1.

**Table 2:** Reliability and validity.

Constructs	Cronbach’s α	CR	AVE
SIC	0.921	0.921	0.594
JC	0.969	0.969	0.673
CSE	0.959	0.959	0.608
TIB	0.886	0.886	0.608

SIC, school innovation climate; JC, job crafting; CSE, creative self-efficacy; TIB, teacher innovation behavior.

### 4.2 Discriminant validity assessment

Discriminant validity was further assessed using the Fornell-Larcker criterion (Fornell and Larcker, 1981). The square roots of AVE for SIC, JC, CSE, and TIB were 0.771, 0.820, 0.780, and 0.780, respectively. Each value exceeded the corresponding inter-construct correlations, which ranged from 0.589 to 0.678. These results support adequate discriminant validity among the four constructs (Table 3).

**Table 3:** Discriminant validity based on the Fornell-Larcker criterion.

Constructs	SIC	JC	CSE	TIB
SIC	**0.771**			
JC	0.639	**0.820**		
CSE	0.600	0.621	**0.780**	
TIB	0.589	0.678	0.627	**0.780**

Diagonal values are the square roots of AVE; off-diagonal values are inter-construct correlations; SIC, school innovation climate; JC, job crafting; CSE, creative self-efficacy; TIB, teacher innovation behavior. The square root of the AVE shown as the bolded diagonal values in the table.

### 4.3 Descriptive statistics and correlation test

Table 4 presents the descriptive statistics and correlation results for the key variables included in this study. The mean (M), standard deviation (SD), skewness (SK), and kurtosis (Kur) were calculated at the composite-score level for each construct, consistent with the composite scores used in the subsequent correlation and regression-based analyses. The composite-score skewness and kurtosis values for all constructs fell within acceptable ranges, indicating no substantial departure from univariate normality at the construct-score level. Regarding the correlation results, school innovation climate is significantly and positively correlated with teacher innovation behavior, job crafting, and creative self-efficacy. Teacher innovation behavior is significantly and positively correlated with job crafting and creative self-efficacy. Job crafting is significantly and positively correlated with creative self-efficacy.

**Table 4:** The description and correlation of variables.

Constructs	*M ± SD*	SK	Kur	SIC	JC	CSE	TIB
SIC	3.041 ± 1.136	−0.109	−1.434	1			
JC	3.520 ± 1.440	0.021	−1.545	0.639***	1		
CSE	3.046 ± 1.122	−0.047	−1.425	0.600***	0.621***	1	
TIB	3.108 ± 1.183	−0.174	−1.386	0.589***	0.678***	0.627***	1

***p < 0.001; SIC, school innovation climate; JC, job crafting; CSE, creative self-efficacy; TIB, teacher innovation behavior.

### 4.4 Common method bias

To examine the potential influence of common method bias (CMB), this study applied Harman’s single-factor test. The results showed that the variance explained by the first factor was 48.905%, which did not exceed the 50% criterion suggested by Podsakoff et al. (2003). This finding indicated that no single factor accounted for most of the variance. Nevertheless, because Harman’s one-factor test serves only as a preliminary diagnostic, these results alone cannot completely exclude the possibility of common method variance.

A confirmatory factor analysis (CFA) was then performed to assess the structural validity of the measurement model. As shown in Table 5, the model fit was within an acceptable range, with *χ^2^* = 1189.251, *df* = 854, *χ^2^/df* = 1.393, RMSEA = 0.026, SRMR = 0.025, GFI = 0.917, AGFI = 0.908, CFI = 0.984, IFI = 0.984, and TLI = 0.983. These indicators met the commonly accepted standards for structural equation modeling, supporting the adequacy of the measurement model. Furthermore, to test for potential common method bias, an unmeasured latent method construct (ULMC) model including a common method factor was established. Compared with the baseline model, the fit indices changed only marginally. Taken together, these analyses did not indicate a dominant common-method factor; however, common method variance cannot be completely ruled out because all focal variables were obtained from the same respondents at a single time point (Marsh and Hocevar, 1985).

**Table 5:** CFA fit indices and comparison between baseline and ULMC models.

Model	*χ^2^(df)*	*χ^2^/df*	RMSEA	SRMR	GFI	AGFI	CFI	IFI	TLI
Reference value		<3	<0.050	<0.090	>0.900	>0.900	>0.900	>0.900	>0.900
Baseline model	1189.251 (854)	1.393	0.026	0.025	0.917	0.908	0.984	0.984	0.983
Method factor model	1161.542 (853)	1.362	0.024	0.025	0.919	0.910	0.985	0.985	0.984
Model Fit Difference			∆RMSEA	∆SRMR	∆GFI	∆AGFI	∆CFI	∆IFI	∆TLI
			0.002	0.000	0.002	0.002	0.001	0.001	0.001
Evaluation criterion			<0.05	<0.05	<0.01	<0.01	<0.01	<0.01	<0.01

df, degree of freedom; RMSEA, root mean square error of approximation; SRMR, standardized root mean square residual; GFI, goodness-of-fit index; AGFI, adjusted goodness-of-fit index; CFI, comparative fit index; IFI, incremental fit index; TLI, tucker-lewis index.

### 4.5 Collinearity test

To assess the robustness of the model and the validity of the subsequent interpretations, collinearity diagnostics were conducted for the predictor variables. Specifically, tolerance and variance inflation factor (VIF) statistics were obtained using SPSS to examine possible multicollinearity. As shown in Table 6, the tolerance values for all variables were higher than 0.10, and the VIF values ranged from 1.838 to 1.985. Since tolerance values above 0.10 indicate no serious collinearity issue, and VIF values below 3.3 are generally considered acceptable (Hair et al., 2019), multicollinearity was not regarded as a major concern in this study.

**Table 6:** Collinearity test of the structural model.

Constructs	Tolerance	VIF
SIC	0.524	1.907
JC	0.504	1.985
CSE	0.544	1.838

SIC, school innovation climate; JC, job crafting; CSE, creative self-efficacy.

### 4.6 Analysis of mediating effects

Table 7 shows that, after controlling for demographic factors such as gender, professional category, and educational background, school innovation climate had a significant positive relationship with job crafting. It was also positively related to creative self-efficacy, while job crafting showed a significant positive association with creative self-efficacy. In addition, teacher innovation behavior was positively associated with school innovation climate, job crafting, and creative self-efficacy, thereby supporting H1.

**Table 7:** Regression analysis of mediator variables.

Result variable	Predictor variable	*B*	*SE*	*t*	Bootstrap 95% CI		*R^2^*	*F*
					LLCI	ULCI		
JC	SIC	0.798	0.040	19.751***	0.719	0.877	0.412	104.857
CSE	SIC	0.167	0.020	8.469***	0.128	0.205	0.451	98.148
	JC	0.159	0.016	10.255***	0.128	0.189		
TIB	SIC	0.171	0.040	4.289***	0.092	0.249	0.546	119.400
	JC	0.326	0.032	10.151***	0.263	0.389		
	CSE	0.585	0.078	7.482***	0.431	0.738		

The reported regression coefficients (B) are unstandardized; ***p < 0.001; SIC, school innovation climate; JC, job crafting; CSE, creative self-efficacy; TIB, teacher innovation behavior.

The mediation results, presented in Table 8 and Figure 2, showed that the total effect estimate for the association between school innovation climate and teacher innovation behavior was 0.602, while the direct effect estimate was 0.171 (95% CI [0.092, 0.249]). The indirect effect through job crafting was 0.260 (95% CI [0.183, 0.346]), accounting for 43.163% of the total effect, indicating a significant indirect association through job crafting between school innovation climate and teacher innovation behavior. The indirect effect through creative self-efficacy was 0.097 (95% CI [0.055, 0.146]), accounting for 16.182% of the total effect, indicating a significant indirect association through creative self-efficacy between school innovation climate and teacher innovation behavior. The sequential mediating effect of job crafting and creative self-efficacy was 0.074 (95% CI [0.044, 0.107]), accounting for 12.311% of the total effect. Because the bootstrap confidence interval for this sequential indirect effect did not include zero, the observed associations were statistically consistent with the proposed sequential mediation pathway from school innovation climate to teacher innovation behavior through job crafting and creative self-efficacy. Therefore, Hypotheses H2, H3, and H4 were supported.

**Table 8:** Chain mediation of job crafting and creative self-efficacy between school innovation climate and teacher innovation behavior.

Effect	Path	*B*	Bootstrap 95% CI		Percentage (%)
			LLCI	ULCI	
Total effect	SIC→TIB	0.602	0.533	0.670	100.00
Direct effect	SIC→TIB	0.171	0.092	0.249	28.327
Indirect effect	SIC→JC → TIB	0.260	0.183	0.346	43.163
	SIC→CSE → TIB	0.097	0.055	0.146	16.182
	SIC→JC → CSE → TIB	0.074	0.044	0.107	12.311

The reported regression coefficients (B) are unstandardized; SIC, school innovation climate; JC, job crafting; CSE, creative self-efficacy; TIB, teacher innovation behavior.

**Figure 2:**
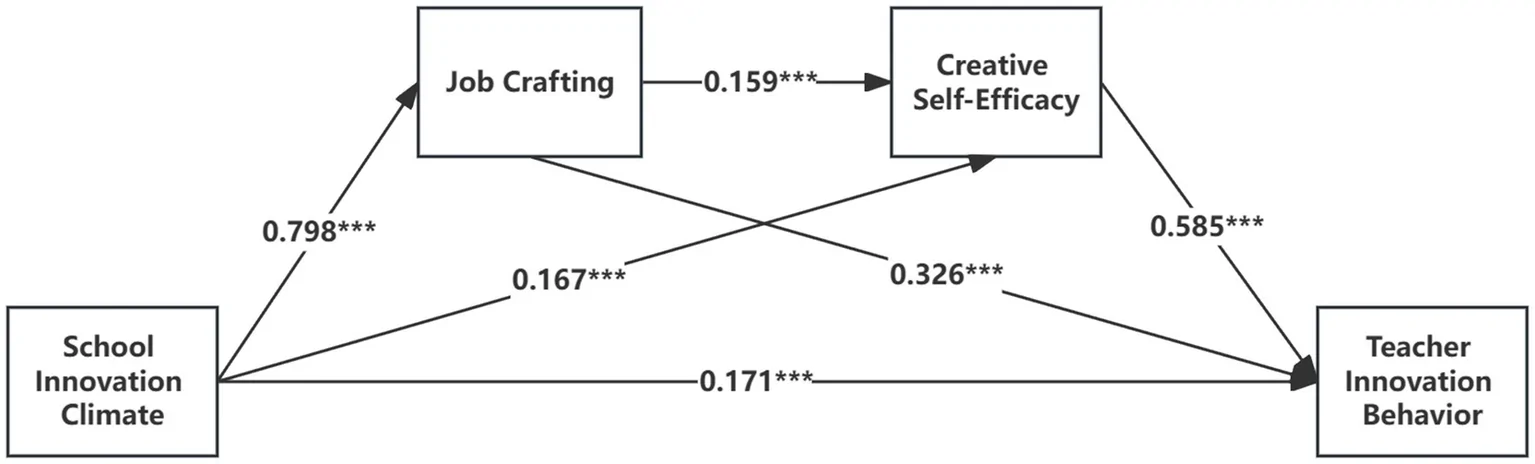
Mediation model.

As a sensitivity analysis, the proposed serial mediation model was additionally examined using latent-variable SEM in AMOS, and the results were substantively consistent with those obtained from the composite-score PROCESS analysis (see Supplementary Tables S2, S3).

## 5 Discussion

With instructors from polytechnic institutions as the sample, this study analyzed how school innovation climate is linked to teacher innovation behavior, and also tested the chain indirect pathways of job crafting and creative self-efficacy. Overall, the empirical results were largely in line with the proposed theoretical assumptions. Specifically, school innovation climate showed a significant positive association with innovation behavior among higher vocational teachers. In addition, job crafting and creative self-efficacy together constituted a serial indirect pathway that connects school innovation climate with teacher innovation behavior. The implications of these findings and the possible psychological pathways underlying the observed associations are discussed below in conjunction with theoretical perspectives and empirical data.

### 5.1 School innovation climate and teacher innovation behavior

The results confirm H1 by showing a robust positive relationship between teacher innovation behavior in higher vocational colleges and school innovation climate. This result aligns with prior studies indicating that educators tend to employ innovative pedagogical methods more actively under conditions where schools support novelty (Al-Mahdy and Elwakil, 2026; Anderson and West, 1998; Bao, 2024). In the JD-R model, school innovation climate is viewed as an organizational-level job resource that provides necessary environmental support for teachers to carry out teaching reform, try new methods, and integrate teaching resources (Bakker and Demerouti, 2007). Within China’s higher vocational education system, teachers are required not only to conduct classroom teaching but also to respond to various demands, such as industry-education integration, skilled talent development, and digital transformation in teaching (Ma et al., 2025; Zhu et al., 2025). An innovation-supportive school climate may be associated with higher vocational teachers’ perceptions of instructional innovation as both practically feasible and institutionally encouraged, as well as with greater openness to experimenting with novel teaching strategies and techniques in the classroom. They might therefore be more open to experimenting with novel teaching strategies and techniques in the classroom. A supportive school innovation climate represents an important contextual factor associated with teacher innovation behavior in higher vocational colleges.

### 5.2 The mediating role of job crafting and creative self-efficacy

The findings support H2 and H3, with significant indirect associations observed through job crafting and creative self-efficacy, respectively, between school innovation climate and teacher innovation behavior among higher vocational teachers. This conclusion is consistent with previous research. For instance, Huang et al. (2022) reported a significant positive association between teachers’ job crafting and creative teaching performance, suggesting that teachers who report greater adjustments to their teaching tasks, work resources, and instructional approaches also tend to report greater use of creative teaching methods. Similarly, Lin and Chiou (2008) and Shi et al. (2023) found that stronger creative self-efficacy is linked with greater teacher involvement in innovative curriculum development and creative instructional practices.

In accordance with the JD-R model, school innovation climate, as an organizational-level work resource, is characterized by resource support, peer collaboration, and environmental conditions conducive to innovation attempts and is positively associated with teachers’ proactive work adjustments and personal psychological resources (Bakker and Demerouti, 2007, 2017). Within this theoretical framework, school innovation climate is associated with a stronger tendency for teachers to engage in job crafting, including a greater willingness to optimize teaching arrangements, integrate teaching resources, and expand professional collaboration. It is also associated with higher creative self-efficacy, including greater confidence in designing innovative courses, adopting new teaching methods, and coping with uncertainties in teaching reform.

Within the context of Chinese higher vocational education, teacher innovation behavior requires continuous coordination among curriculum objectives, classroom activities, student ability differences, and instructional resource conditions (Ma et al., 2025; Zhu et al., 2025). In these complex teaching contexts, the association between school-level innovation support and teacher innovation behavior may coexist with teachers’ job crafting and creative self-efficacy. Teachers also need to proactively adjust teaching tasks, reconceptualize the value of their teaching work, and develop confidence in their own creative teaching abilities in practice. Therefore, a positive school innovation climate may be positively associated with both teachers’ job crafting and creative self-efficacy. Specifically, stronger perceptions of school innovation climate may coexist with greater willingness to optimize teaching arrangements, integrate instructional resources, and expand professional collaboration, as well as greater confidence in designing innovative curricula, adopting novel teaching methods, and coping with uncertainties in teaching reform.

### 5.3 The chain mediating effect of job crafting and creative self-efficacy

The findings support H4, as the observed associations were consistent with the proposed sequential mediation pathway involving school innovation climate, job crafting, creative self-efficacy, and teacher innovation behavior in higher vocational colleges. This result is in line with earlier studies (Huang et al., 2022; Huang et al., 2023; Shi et al., 2023), suggesting that school innovation climate is associated with teachers’ job crafting, which is in turn associated with creative self-efficacy, while these constructs are also positively associated with teacher innovation behavior. In the JD-R model, school innovation climate, as an organizational-level job resource, provides a supportive environment for teachers to carry out teaching reform and is associated with a tendency to proactively optimize teaching practice (Bakker and Demerouti, 2007, 2017). Teachers’ engagement in redesigning teaching activities, integrating instructional resources, and expanding professional collaboration may be associated with successful experiences, positive feedback, and greater confidence in conducting innovative teaching activities. Within Chinese higher vocational education, teachers’ teaching innovation is generally characterized by a strong application-oriented focus. The implementation of innovative plans is not solely a matter of resources provided by schools; it also involves teachers’ proactive conversion of these resources into specific classroom designs, practical training organization, and teaching interactions (Ma et al., 2025; Zhu et al., 2025). Thus, school innovation climate is associated with teacher innovation behavior both directly and indirectly through its statistical associations with job crafting and creative self-efficacy. Overall, this study identifies a theoretically meaningful pattern of associations among school innovation climate, job crafting, creative self-efficacy, and teacher innovation behavior among higher vocational teachers. Specifically, teachers’ proactive behaviors may represent an important link between organizational resources and personal psychological resources, both of which are associated with teacher innovation behavior.

## 6 Implications

### 6.1 Theoretical implications

This study has the following theoretical implications. First, our research substantiates the relationship between teachers’ inventive conduct in the setting of vocational education and the school innovation climate by concentrating on instructors in higher vocational institutions. The results indicate that school innovation climate is not only an organizational environmental factor in the general sense, but also an important contextual condition for teachers to engage in teaching reform and practical innovation within vocational education. With specific reference to the features of vocational education, this study contributes to our knowledge of the contextual underpinnings of teacher innovation behavior at higher vocational institutions.

Second, building on the JD-R model, this study integrated school innovation climate, job crafting, creative self-efficacy, and teacher innovation behavior into one analytical framework. In doing so, it offers new empirical evidence for applying the JD-R model to educational settings, especially among higher vocational teachers. The findings imply that teacher innovation behavior is linked to organizational work resources both directly and indirectly through their proactive behavior and personal psychological resources. This further extends the explanatory value of the JD-R model in studies of teacher innovation behavior.

Lastly, this study identified a pattern of sequential indirect associations among school innovation climate, job crafting, creative self-efficacy, and teacher innovation behavior. These results provide further insight into the organizational, behavioral, and psychological factors associated with teacher innovation behavior in higher vocational colleges. The finding also indicates that school-level innovation support is directly associated with teacher innovation behavior, and indirectly associated with it through job crafting and creative self-efficacy. This provides a more detailed theoretical explanation for the mechanisms through which organizational contextual resources are associated with teacher innovation behavior.

### 6.2 Practical implications

The study’s conclusions have useful ramifications for higher vocational schools looking to encourage innovative teaching practices. First, higher vocational colleges should cultivate a school climate supportive of teaching innovation, taking into account the applied and practice-oriented characteristics of vocational education. Specifically, institutions need to refine teaching reform policies, provide adequate practical training platforms and curriculum resources, and support the implementation of industry-education integration and project-based instruction. These measures may provide a more supportive context for instructional experimentation and may be associated with stronger perceptions of institutional and resource support among teachers.

Second, higher vocational colleges should place importance on the cultivation of teachers’ job crafting abilities. Schools can leverage course teams, professional group construction, and school-enterprise cooperation projects to encourage teachers to proactively adjust teaching tasks, optimize practical teaching methods, and expand professional interactions with peers, enterprise mentors, and students. Sustained work adjustments may coexist with stronger perceptions of organizational support and greater internal motivation for teaching innovation.

Finally, higher vocational colleges should attend to the enhancement of teachers’ creative self-efficacy. Demonstration courses, sharing of exemplary cases, peer feedback mechanisms, and enterprise practice support may provide supportive contexts associated with greater creative self-efficacy and greater confidence in addressing challenges in teaching reform. As a key psychological resource, creative self-efficacy is closely associated with teachers’ perceptions of external support and intrinsic motivation for sustained innovation.

Overall, teaching innovation by higher vocational teachers requires the synergy of school support, proactive teacher adjustments, and creative teaching beliefs. College administrators should recognize that teacher innovation behavior is associated with the joint presence of organizational resources, proactive teacher behaviors, and personal psychological resources.

### 6.3 Limitations and future directions

There are a number of limitations to this study.

First, the sample was drawn from three higher vocational colleges in Henan Province. Although teachers were randomly selected from institutional rosters within the participating colleges, the institutions themselves were not sampled to represent the national population of higher vocational colleges. In addition, teachers aged 51 years or above represented 33.2% of the analytic sample, which may reflect institution-specific staffing characteristics or possible sampling imbalance. Because some of the excluded questionnaires contained incomplete demographic information, a systematic statistical comparison between the excluded and retained cases across the observed demographic characteristics could not be conducted. Therefore, potential selection bias associated with case exclusion cannot be completely ruled out. The findings should be generalized beyond the participating institutions and Henan Province with caution. Future studies should recruit teachers from multiple provinces and different types of higher vocational colleges and employ stratified or multi-stage sampling designs to improve sample representativeness and the generalizability of the findings.

Second, although the present study positioned job crafting before creative self-efficacy based on the theoretical distinction between proactive work adjustment and teachers’ beliefs about their creative capabilities, the cross-sectional design cannot establish the temporal precedence of these two constructs. Therefore, the possibility that creative self-efficacy may precede job crafting cannot be excluded. Teachers with stronger confidence in their creative teaching capabilities may also be more willing to proactively adjust their teaching tasks, work cognition, and professional relationships. The relationship between job crafting and creative self-efficacy may therefore be more complex than the sequential ordering examined in the present study. Future longitudinal or multi-wave studies should compare alternative temporal orderings between job crafting and creative self-efficacy and examine whether their relationship may change or operate reciprocally over time.

Third, in order to identify links between school innovation climate, job crafting, creative self-efficacy, and teacher innovation behavior, this study first used a cross-sectional questionnaire methodology. Because of this, it is challenging to document how these variables dynamically alter over time. To further understand the temporal ordering and longitudinal associations among school innovation climate, job crafting, creative self-efficacy, and teacher innovation behavior, future studies might include multi-wave research designs or longitudinal monitoring.

Fourth, all focal variables in this study were measured using teacher self-report scales at a single time point. Although Harman’s single-factor test and a ULMC analysis were conducted, and the first factor accounted for 48.905% of the variance, which was below the reference threshold, common method bias and subjective evaluation biases, such as social desirability, cannot be completely ruled out because all variables were based on self-reports from the same source. Future research should adopt time-lagged and multi-source designs, incorporating student or peer evaluations, classroom observations, and objective teaching outcome indicators, to reduce shared-source bias and provide more robust evidence of teacher innovation behavior.

Finally, this study focused primarily on three factors—school innovation climate, job crafting, and creative self-efficacy—without incorporating other potentially relevant variables such as teachers’ digital literacy, professional development opportunities, organizational support, or leadership styles. Future research could further expand the model and examine differences among different types of higher vocational colleges, professional categories, or teachers with varying professional titles, thereby gaining a more comprehensive understanding of the conditions under which higher vocational teacher innovation behavior emerges.

## 7 Conclusion

Grounded in the JD-R model, our study explored how school innovation climate relates to teacher innovation behavior in higher vocational colleges, with particular attention to the sequential mediating effects of job crafting and creative self-efficacy. The findings showed a strong positive linkage between higher vocational instructors’ inventive activity and the school’s innovation climate. Moreover, significant indirect associations were observed through job crafting and creative self-efficacy separately, and the findings were also consistent with the proposed sequential mediation pathway involving these two variables. The findings suggest that organizational resources, teachers’ proactive work adjustments, and individual psychological resources are all meaningfully associated with teacher innovation behavior among higher vocational teachers. This study therefore offers new empirical evidence for understanding the factors and sequential associations related to teacher innovation behavior. It also provides practical implications for higher vocational colleges, suggesting that teaching innovation may be supported in contexts characterized by an innovation-supportive environment, greater job crafting, and stronger creative self-efficacy. However, our study used a cross-sectional questionnaire design. Future research could further examine the temporal ordering and longitudinal associations among school innovation climate, job crafting, creative self-efficacy, and teacher innovation behavior by integrating longitudinal tracking, classroom observation, or multi-source data.

## Data Availability

The raw data supporting the conclusions of this article will be made available by the authors, without undue reservation.
