# Supplementary material for: The relationship between school innovation climate and teacher innovation behavior in higher vocational colleges: the chain mediating role of teacher job crafting and creative self-efficacy
Source: Front Psychol. 2026 Sep 14;17:1924843. doi: 10.3389/fpsyg.2026.1924843 (PMC13616942; doi:10.3389/fpsyg.2026.1924843)
Supplement: Supplementary file 1 [file Supplementary_file_1.docx]

**Supplementary Material**

**Manuscript title:**

The Relationship Between School Innovation Climate and Teacher Innovation Behavior in Higher Vocational Colleges: The Chain Mediating Role of Teacher Job Crafting and Creative Self-Efficacy

Table S1. Factor Loadings, CR, and AVE

| Construct | Item | Loading | CR | AVE |
| --- | --- | --- | --- | --- |
| SIC | SIC1 | 0.753 | 0.921 | 0.594 |
|  | SIC2 | 0.762 |  |  |
|  | SIC3 | 0.753 |  |  |
|  | SIC4 | 0.790 |  |  |
|  | SIC5 | 0.746 |  |  |
|  | SIC6 | 0.779 |  |  |
|  | SIC7 | 0.793 |  |  |
|  | SIC8 | 0.790 |  |  |
| JC | JC1 | 0.829 | 0.969 | 0.673 |
|  | JC2 | 0.842 |  |  |
|  | JC3 | 0.818 |  |  |
|  | JC4 | 0.824 |  |  |
|  | JC5 | 0.830 |  |  |
|  | JC6 | 0.818 |  |  |
|  | JC7 | 0.844 |  |  |
|  | JC8 | 0.802 |  |  |
|  | JC9 | 0.817 |  |  |
|  | JC10 | 0.799 |  |  |
|  | JC11 | 0.855 |  |  |
|  | JC12 | 0.801 |  |  |
|  | JC13 | 0.831 |  |  |
|  | JC14 | 0.831 |  |  |
|  | JC15 | 0.757 |  |  |
| CSE | CSE1 | 0.750 | 0.959 | 0.608 |
|  | CSE2 | 0.796 |  |  |
|  | CSE3 | 0.795 |  |  |
|  | CSE4 | 0.768 |  |  |
|  | CSE5 | 0.800 |  |  |
|  | CSE6 | 0.779 |  |  |
|  | CSE7 | 0.781 |  |  |
|  | CSE8 | 0.761 |  |  |
|  | CSE9 | 0.806 |  |  |
|  | CSE10 | 0.777 |  |  |
|  | CSE11 | 0.794 |  |  |
|  | CSE12 | 0.784 |  |  |
|  | CSE13 | 0.778 |  |  |
|  | CSE14 | 0.774 |  |  |
|  | CSE15 | 0.746 |  |  |
| TIB | TIB1 | 0.773 | 0.886 | 0.608 |
|  | TIB2 | 0.762 |  |  |
|  | TIB3 | 0.772 |  |  |
|  | TIB4 | 0.789 |  |  |
|  | TIB5 | 0.803 |  |  |

*Note. SIC: school innovation climate; JC: job crafting; CSE: creative self-efficacy; TIB: teacher innovation behavior.*

Table S2. Structural Path Estimates of the Latent-Variable SEM Sensitivity Analysis

| Result variable | Predictor variable | *B* | *p* | Bootstrap 95% CI | |
| --- | --- | --- | --- | --- | --- |
|  |  |  |  | LLCI | ULCI |
| JC | SIC | 0.854 | < 0.001 | 0.748 | 0.967 |
| CSE | SIC | 0.356 | < 0.001 | 0.246 | 0.475 |
|  | JC | 0.292 | < 0.001 | 0.206 | 0.386 |
| TIB | SIC | 0.177 | 0.009 | 0.047 | 0.323 |
|  | JC | 0.328 | < 0.001 | 0.219 | 0.442 |
|  | CSE | 0.312 | < 0.001 | 0.183 | 0.437 |

*Note. The reported regression coefficients (B) are unstandardized; SIC: school innovation climate; JC: job crafting; CSE: creative self-efficacy; TIB: teacher innovation behavior.*

Table S3. Latent-Variable SEM Estimates

| Effect | Path | *B* | Bootstrap 95% CI | | Percentage (%) |
| --- | --- | --- | --- | --- | --- |
|  |  |  | LLCI | ULCI |  |
| Total effect | SIC→TIB | 0.646 | 0.552 | 0.749 | 100.00 |
| Direct effect | SIC→TIB | 0.177 | 0.047 | 0.323 | 27.399 |
| Indirect effect | SIC→JC→TIB | 0.280 | 0.188 | 0.386 | 43.344 |
|  | SIC→CSE→TIB | 0.111 | 0.061 | 0.179 | 17.183 |
|  | SIC→JC→CSE→TIB | 0.078 | 0.045 | 0.122 | 12.074 |

*Note. The reported regression coefficients (B) are unstandardized; SIC: school innovation climate; JC: job crafting; CSE: creative self-efficacy; TIB: teacher innovation behavior.*
